# Oncologic outcomes of calcitonin-negative medullary thyroid carcinoma

**DOI:** 10.3389/fendo.2022.1025629

**Published:** 2022-11-23

**Authors:** Wenliang Yue, Yong Zhang

**Affiliations:** Department of Thyroid and Breast Surgery, Anyang People Hospital, Anyang, Henan, China

**Keywords:** calcitonin, medullary thyroid carcinoma, calcitonin-negative medullary thyroid carcinoma, biochemical cure, neuroendocrine tumor

## Abstract

**Objective:**

Calcitonin (Ct)-negative medullary thyroid carcinoma (MTC) is a rare neuroendocrine tumor. This study aimed to clarify its incidence, clinicopathologic characteristics, management, and treatment outcome.

**Methods:**

We retrospectively analyzed data of patients with primary MTC. Patients were divided into two groups according to the preoperative serum Ct level (Ct-negative and Ct-positive). The demographic, pathologic, and molecular characteristics, and treatment outcomes were compared between the two groups. In the Ct-negative group, we analyzed the association between the operation type and treatment outcome.

**Results:**

Of the total 312 patients, 24 were diagnosed with Ct-negative MTC. The rate of lymph node metastasis was significantly higher in the Ct-positive than in the Ct-negative group (47.9% vs. 0%, *p*<0.001). The proportion of patients with Ki-67 ≤10% was significantly higher in the Ct-negative than in the Ct-positive group (87.5% vs. 38.2%, *p*<0.001). Excellent response was achieved by 91.7% and 34.7% of patients in the Ct-negative and Ct-positive groups, respectively (*p*<0.001). In the Ct-negative group, excellent response was achieved by all female patients, but only 50% of male patients.

**Conclusions:**

Ct-negative MTC is rare and unlikely to develop lymph node metastasis. Unilateral lobectomy tends to provide a satisfactory chance of excellent response; however, this requires further validation.

## Introduction

Medullary thyroid carcinoma (MTC), a malignant tumor arising from thyroid parafollicular C-cells characterized by the production of calcitonin (Ct), accounts for approximately 5% of all thyroid cancers (1). It can develop as a sporadic or hereditary familial tumor (2). Although sporadic MTC is usually well differentiated, less aggressive, and has a low growth rate (3), it has unpredictable outcomes. Complete tumor excision is currently the only curative therapy; thus, early diagnosis of MTC is essential (4).

Although elevated serum Ct level can be found in hyperparathyroidism, small/large-cell lung cancer, prostate cancer, renal failure, and other conditions, it is considered a reliable biomarker for MTC diagnosis, prognosis, and recurrence (5). Usually, there is a direct relationship between serum Ct levels and tumor size (6); however, since the first report of an MTC with normal serum Ct levels (7), a few similar cases (less than 50) have been described (8–12).

Due to the rarity of Ct-negative MTC, its incidence, clinicopathologic characteristics, management, and prognosis remain unknown. Therefore, the current study aimed to clarify these questions to contribute to better understanding and improved treatment of this disease in the future.

## Patients and methods

### Ethical considerations

The institutional research committee approved this study. All patients provided written informed consent for all diagnostic and treatment procedures. The requirement for informed consent for participation in this study was waived due to the retrospective study design. All procedures performed in this study were in accordance with the 1964 Helsinki Declaration and its later amendments or comparable ethical standards.

### Patient selection

**Figure 1:**
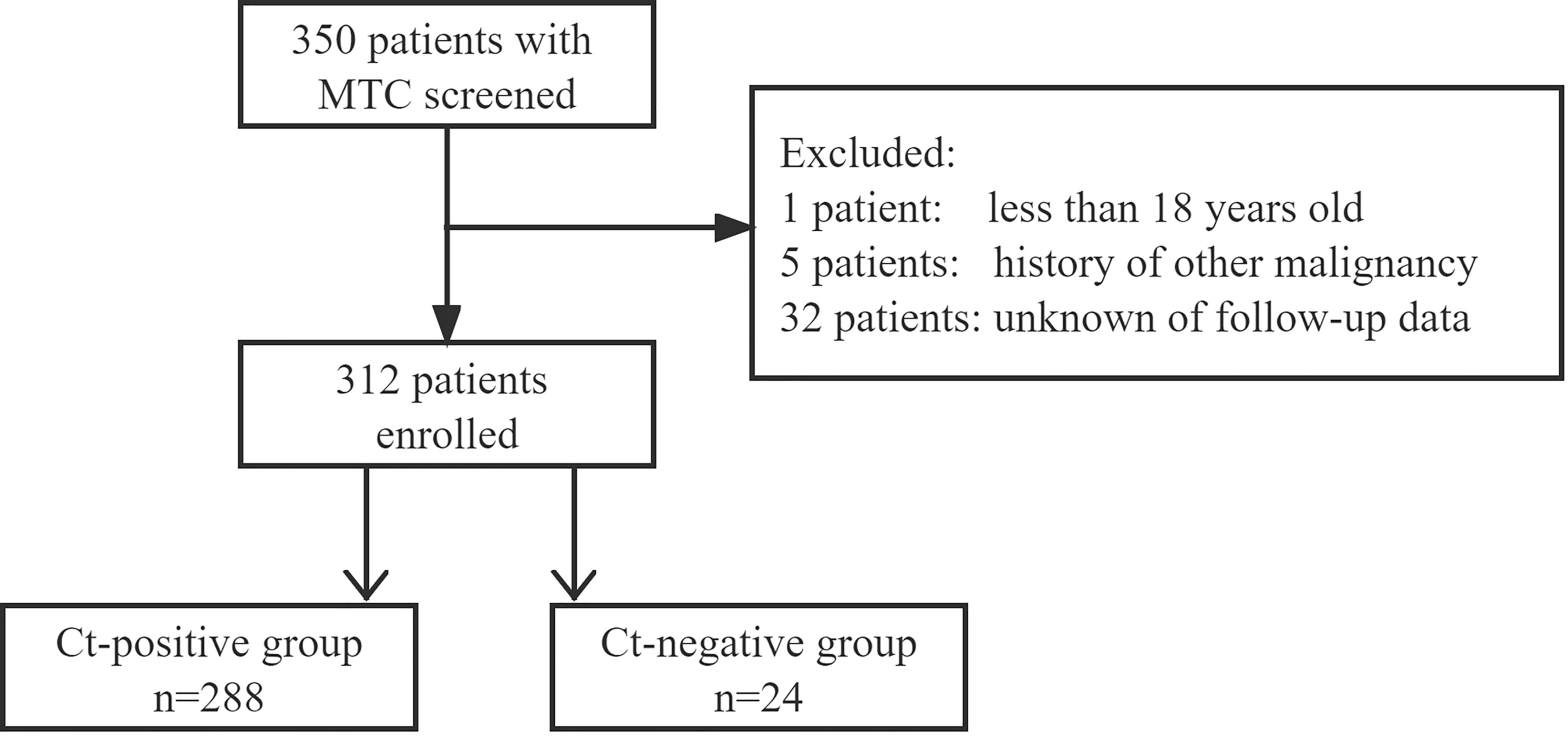
Study flowchart. Ct, calcitonin; MTC, medullary thyroid carcinoma.

We retrospectively reviewed the medical records of patients with surgically treated sporadic or hereditary MTC in a tertiary medical center from January 2010 to July 2022. The inclusion criteria were as follows: age > 18 years; primary disease; and available follow-up data. Patients with a history of other malignancies were excluded (**Figure 1**). Information on demography, pathology, treatment, and follow-up was extracted and analyzed.

### Biochemical testing

Serum Ct level was measured in all patients with suspected thyroid malignant nodules. The blood sample was drawn in the morning after overnight fasting and sent to the laboratory immediately (13). Measurement was performed using the chemiluminescent immunometric assay, and normal ranges were 0−6.4 pg/mL for women and 0−9.52 pg/mL for men. MTC with a serum Ct level of < 6.4 pg/mL in women or < 9.52 pg/mL in men was considered to be Ct-negative.

Serum carcinoembryonic antigen (CEA) levels were also measured. The normal range of CEA was 0−4.7 ng/mL.

### Treatment and follow-up

In our center, fine needle aspiration is selectively used, particularly for nodules less than 10 mm in size. If MTC was diagnosed based on fine needle aspiration or serum Ct level or intraoperative frozen section, total thyroidectomy with central neck dissection was performed. Therapeutic lateral neck dissection (II−V) was implemented if there was lateral neck lymph node metastasis pathologically (14, 15). If MTC was diagnosed based on the postoperative pathologic findings, the decision on reoperation was made in consideration of the patient’s willingness, tumor’s characteristics, and surgeon’s preference (16).

RET gene mutation analysis was performed by polymerase chain reaction testing, as described previously (4, 8, 9), using the TaKaRa Ex Taq kit and ABI Prism 3100 Genetic Analyzer. Immunohistochemical analysis for CEA, chromogranin A (CgA), Ct, and Ki-67 was performed for all patients.

Patients were followed up every 3−6 months for the first 2 years and every 6−12 months thereafter. Serum Ct and CEA level assessment, as well as thyroid gland ultrasound or enhanced computed tomography were performed on each follow-up visit. If there was doubt of disease recurrence, needle biopsy was usually performed to confirm the diagnosis. Reoperation was the first-choice treatment for recurrent foci.

### Statistical analysis

Patients were divided into two groups according to the preoperative serum Ct level (Ct-negative vs. Ct-positive). Treatment outcome was classified as excellent or non-excellent response. Excellent response was defined as a status of undetectable Ct and no evidence of structural disease (16). The Chi-square test was used to compare the demographic, pathologic, and molecular characteristics, and treatment outcomes between the two groups, and to analyze the association between the operation type and treatment outcome in the Ct-negative group. All statistical analyses were performed using IBM SPSS Statistics 20.0 (IBM Corp., Armonk, NY, U.S.A.). Statistical significance was set at *p*<0.05.

## Results

A total of 312 patients were enrolled, 288 (219 women and 69 men) with a mean age of 50.7 ± 13.5 years in the Ct-positive group, and 24 (20 women and 4 men) with a mean age of 51.3 ± 9.0 years in the Ct-negative group.

The mean tumor size was 19.4 ± 12.5 mm in the Ct-positive and 15.8 ± 8.9 mm in the Ct-negative group. In the Ct-positive group, multiple tumor foci were detected in 20 patients, and papillary thyroid carcinoma coexisted in 30 patients. In the Ct-negative group, all patients had only one focus, and there were no cases of concurrent papillary thyroid carcinoma or other malignant tumors. While there were six cases of somatic RET gene mutations, with one case of familial MTC in the Ct-positive group, neither RET gene mutation nor hereditary familial MTC was detected in the Ct-negative group.

There was a statistically significant difference between the groups in the extent of surgery, with the proportion of patients undergoing extensive surgery being greater in the Ct-positive than in the Ct-negative group (*p*<0.001). In the Ct-positive group, all patients underwent total thyroidectomy with central neck dissection, and 88 patients also required lateral neck dissection. In the Ct-negative group, 17 patients underwent unilateral lobectomy with central neck dissection and seven patients underwent total thyroidectomy with central neck dissection. Central and lateral neck lymph node metastasis was present in 138 and 88 patients in the Ct-positive group, respectively, but in none of the patients in the Ct-negative group. The Ct-negative group had significantly lower lymph node metastasis rate (0% vs. 47.9%) and serum CEA level than the Ct-positive group (*p*<0.001 for both). There were no significant differences between the two groups regarding age, sex, tumor size, and other baseline parameters (all *p*>0.05, **Table 1**).

**Table 1:** Comparison of clinicopathologic variables between negative and positive groups.

Variable	Serum calcitonin level		p
	Positive (n = 288)	Negative (n = 24)	
Age			
≤50	149	12	
>50	139	12	0.870
Sex			
Male	69	4	
Female	219	20	0.418
Tumor size (mm)			
≤10	78	8	
>10	210	16	0.510
Serum Carcino-Embryonic Antigen level			
Normal	8	22	
Elevated	280	2	<0.001
RET gene mutation^			
Yes	6	0	
No	105	24	0.370
Foci			
Single	268	24	
Multiple	20	0	0.248
MTC* type			
Sporadic	287	24	
Familial	1	0	1.000
Con occurrence			
PTC^&^	30	0	
No	258	24	0.146
Lymph node metastasis			
Yes	138	0	
No	150	24	<0.001
Operation type^!^			
TT+CLND	200	7	
TT+CLND+LLND	88	0	
UL+CLND	0	17	<0.001

*MTC, Medullary thyroid carcinoma;

^Status of RET gene mutation in 177 patients was unknown;

^&^PTC, Papillary thyroid carcinoma;

^!^TT, total thyroidectomy; CLND, central lymph node dissection; LLND, lateral lymph node dissection; UL, unilateral lobectomy.

In the immunohistochemical analysis, positivity for Ct, CEA, and CgA was detected in 221, 167, and 139 patients in the Ct-positive group, and 14, 10, and seven patients in the Ct-negative group, respectively. There was a significant difference between the groups regarding the proliferative capacity; Ki-67 >10% was detected in 178 patients in the Ct-positive and in only three patients in the Ct-negative group (*p*<0.001, **Table 2**).

**Table 2:** Comparison of immunohistochemistry variables between negative and positive groups.

Variable	Serum calcitonin level		p
	Positive (n = 288)	Negative (n = 24)	
Calcitonin			
Positive	221	14	
Negative	67	10	0.052
Carcino-Embryonic Antigen			
Positive	167	10	
Negative	121	14	0.121
Chromogranin A			
Positive	139	7	
Negative	149	17	0.072
Ki-67			
≤10%	110	21	
>10%	178	3	<0.001

After the initial operation, the rate of excellent response was significantly higher in the Ct-negative than in the Ct-positive group (91.7% vs. 34.7%, *p*<0.001, **Table 3**). Only two patients in the Ct-negative group had a non-excellent response, a 67-year-old female patient with an initial tumor size of 1.0 cm and a 61-year-old female patient with an initial tumor size of 0.6 cm. However, at the last follow-up, their serum Ct levels were 34.2 pg/mL and 11.2 pg/mL, respectively, and neither of them had structurally identifiable disease.

**Table 3:** Comparison of treatment outcome between negative and positive groups.

Outcome	Serum calcitonin level		p
	Positive (n = 288)	Negative (n = 24)	
Excellent response	100	22	
Other	188	2	<0.001

In the subgroup analysis in the Ct-negative group, the excellent response rate was 50% among men and 100% among women, with a statistically significant difference (*p*=0.022). There was no significant difference in the excellent response rate according to the operation type; excellent response was achieved by six of seven patients who underwent total thyroidectomy, and by 16 of 17 patients who underwent unilateral lobectomy (*p*=1.000). Additionally, no significant difference was observed regarding age, tumor size, and serum CEA levels (all *p*>0.05, **Table 4**).

**Table 4:** Association between clinicopathologic factors and treatment outcome in negative group.

Factor	Treatment outcome		p
	Excellent response	Other	
Age			
≤50	11	1	
>50	11	1	1.000
Sex			
Male	2	2	
Female	20	0	0.022
Tumor size (mm)			
≤10	8	0	
>10	14	2	0.536
Serum Carcino-Embryonic Antigen level			
Normal	21	1	
Elevated	1	1	0.163
Operation type			
Total thyroidectomy	6	1	
Unilateral lobectomy	16	1	1.000

## Discussion

This study found that, compared to typical MTC, Ct-negative MTC tended to have limited capacity of lymph node metastasis and proliferation, and a good prognosis independent of the operation extent. However, male patients might require more aggressive treatment.

Ct-negative MTC is a rare disease, most commonly described in case reports (12, 17–20). Hence, its incidence remains unknown. Frank-Raue et al. (11) identified seven nonsecretory MTCs among 839 sporadic cases for an incidence of 0.83%, while Zhou et al. (10) reported Ct-negative MTC in 12.0% of 158 MTC cases. In the current study, the incidence was 7.8%. The wide incidence range might be explained by the different detection reagents and methods. The variable level of different products of the Ct gene results in several circulating immunoreactive isoforms and fragments, giving rise to disparate assay formats and Ct antibody concentrations (13).

The clinicopathologic characteristics of Ct-negative MTC have attracted much attention. Murphy et al. (12) presented a case of MTC in which the tumor size was 35 mm, but RET gene mutation analysis was negative. Similarly, Chernyavsky et al. (19) reported an MTC sized approximately 15 mm, but the RET proto-oncogene mutation assay was negative for mutations in exons 10, 11, 13, 14, 15, and 16. In the three cases reported by Jingzhu et al. (20), the tumor size ranged from 3 to 70 mm, and one patient had three foci. In the case series of Kim et al. (9), the mean tumor size in their 19 patients was only 6.5 mm. Interestingly, our results supported these findings. As the serum Ct level usually increases with tumor size, these findings might indicate that there is an inherent difference between Ct-negative and typical MTC. This difference could be explained by at least four aspects: 1) the mechanism of Ct synthesis may be defective, or alternatively, the storage and/or secreting apparatus could be impaired; 2) a defect in Ct production might be related to dedifferentiation, which does not support the wide range of clinical outcomes in these patients, from long-term survival to rapid progression and death; 3) precursor peptides and aberrant forms of Ct may be produced, resulting in inability to form mature Ct; and 4) serum Ct may degrade rapidly at room temperature owing to the property of high instability (20). Notably, in almost all reported Ct-negative MTC cases, the RET gene mutation test was negative. As this test detects approximately 95% of mutations causative of familial MTC, this finding suggests that Ct-negative MTC is very likely to be sporadic.

CEA is an important tumor marker for gastrointestinal malignancy and MTC. In a recent review, it was found that preoperative CEA values >30 ng/mL were indicative of extra-thyroid disease, while CEA values >100 ng/mL were related to lymph node involvement and distant metastasis. Furthermore, increase in the preoperative CEA values was associated with presence of lymph node metastasis or distant metastasis, larger size of the primary tumor, and a poorer prognosis (21). It has also been shown that serum CEA levels in typical MTC are significantly higher than those in Ct-negative MTC. However, different results have been reported regarding CEA positivity or negativity in immunohistochemistry (9, 10). The current study confirmed these findings; the rate of CEA positivity in the immunohistochemical analysis was similar in Ct-negative and typical MTCs. As MTC is a neuroendocrine tumor, CgA is also a reliable biomarker (22). However, in this study, there was no statistically significant difference between the two groups regarding CgA positivity in immunohistochemistry. Although the two types of MTC might be the same in nature and share common characteristics, they also have distinctive features, as reflected by the significantly lower Ki-67 index in Ct-negative MTCs, which may have contributed to the phenomenon of absent lymph node metastasis.

Excellent treatment response, which was our goal, refers to a status of undetectable Ct and no structural evidence of disease (17). Notably, in the present study, almost all patients in the Ct-negative group achieved excellent response, and the rate was statistically higher than that in the Ct-positive group. This result is consistent with previously reported findings. Namely, the rate of achievement of undetectable Ct was found to be independently affected by lymph node metastasis, tumor stage, and tumor size (23); thus, patients with advanced-stage disease are less likely to achieve this goal. Furthermore, Cohen et al. (24) reported postoperative normalization of Ct levels in 97% of patients when the preoperative level was below 50 pg/mL, but the rate decreased to 41.7% when preoperative Ct levels exceeded 50 pg/mL. Other studies have also reported that undetectable Ct was less likely to be achieved in cases of high preoperative Ct levels (5, 25).

Predictors of excellent response in Ct-negative MTC have not been analyzed previously. In the current study, sex was the only significant factor, and male patients were less likely to achieve excellent response. In a previous study, risk factors preventing achievement of undetectable Ct in typical MTC included extrathyroidal tumor involvement, distant metastasis, and positive lymph node disease, while patients’ sex had minimal influence (26). This discrepancy in the results partially supports the unique features of Ct-negative MTC, which requires further molecular research. Furthermore, although major international guidelines recommend total thyroidectomy for MTC of any size to achieve better oncologic outcomes (2, 27, 28), in the present study, the operation type had no influence on the excellent response rate, suggesting that unilateral lobectomy might be suitable for Ct-negative MTC. However, increased postoperative Ct levels were noted in two patients, one of whom underwent unilateral lobectomy. It has been reported that initially nonsecretory MTC may become secretory MTC regardless of the tumor size (11). Although the underlying mechanism remains unknown, this finding highlights the need for regular Ct and CEA level measurements in the follow-up of patients with Ct-negative MTC.

The current study might be the first to describe the characteristics of Ct-negative MTC. Our findings could provide significant insight into the rational management of this rare disease. Nonetheless, the study also had some limitations. First, as a retrospective study, it had inherent bias; second, our sample size was relatively small, which would have decreased the statistical power; third, the underlying molecular mechanism was not analyzed. Therefore, further basic and clinical research is required.

In summary, Ct-negative MTC is rare and unlikely to develop lymph node metastasis. Unilateral lobectomy tends to provide a satisfactory chance of excellent response; however, this requires further validation.

## Data availability statement

The original contributions presented in the study are included in the article/supplementary material. Further inquiries can be directed to the corresponding author.

## Ethics statement

The studies involving human participants were reviewed and approved by Anyang People Hospital. The patients/participants provided their written informed consent to participate in this study.

## Author contributions

All authors listed have made a substantial, direct, and intellectual contribution to the work and approved it for publication.

## Conflict of interest

The authors declare that the research was conducted in the absence of any commercial or financial relationships that could be construed as a potential conflict of interest.

## Publisher’s note

All claims expressed in this article are solely those of the authors and do not necessarily represent those of their affiliated organizations, or those of the publisher, the editors and the reviewers. Any product that may be evaluated in this article, or claim that may be made by its manufacturer, is not guaranteed or endorsed by the publisher.
